# Network Neuroscience Untethered: Brain-Wide Immediate Early Gene Expression for the Analysis of Functional Connectivity in Freely Behaving Animals

**DOI:** 10.3390/biology12010034

**Published:** 2022-12-24

**Authors:** Dylan J. Terstege, Jonathan R. Epp

**Affiliations:** Department of Cell Biology and Anatomy, Hotchkiss Brain Institute, Cumming School of Medicine, University of Calgary, Calgary, AB T2N 4N1, Canada

**Keywords:** immediate early genes, functional connectivity, animal behaviour, systems neuroscience, brain-wide activity

## Abstract

**Simple Summary:**

Understanding the relationships between sets of regions across the brain is critical to understanding of cognitive function. The application of graph theoretical analyses to brain-wide immediate early gene expression density provides a means of studying these relationships in freely behaving animal models. Here, we provide overviews of the steps required to apply these techniques and acknowledge critical considerations which should be kept in mind when designing and conducting these experiments.

**Abstract:**

Studying how spatially discrete neuroanatomical regions across the brain interact is critical to advancing our understanding of the brain. Traditional neuroimaging techniques have led to many important discoveries about the nature of these interactions, termed functional connectivity. However, in animal models these traditional neuroimaging techniques have generally been limited to anesthetized or head-fixed setups or examination of small subsets of neuroanatomical regions. Using the brain-wide expression density of immediate early genes (IEG), we can assess brain-wide functional connectivity underlying a wide variety of behavioural tasks in freely behaving animal models. Here, we provide an overview of the necessary steps required to perform IEG-based analyses of functional connectivity. We also outline important considerations when designing such experiments and demonstrate the implications of these considerations using an IEG-based network dataset generated for the purpose of this review.

## 1. Introduction

The brain is a complex and dynamic system across which the activities of many neuroanatomical regions fluctuate. These fluctuations form distinct patterns of coactivation, otherwise known as functional connectivity [1]. These analyses most often use techniques such as functional magnetic resonance imaging to infer regional activity throughout the brain in real time [2,3,4]. While functional connectivity is commonly measured in humans it has been less common to do so in animal models. However, similar imaging techniques have been applied in animals but typically require anaesthesia or head fixation [5,6]. More recently, advances in imaging of in vivo calcium or voltage sensors have been recognized as another approach for assessing functional connectivity in awake animal models. Significant advances have been made with these techniques, which can now image thousands of neurons and large volumes of cortex in freely-behaving rodents [7,8]. While these advancements enable exciting new ways to study neuronal population dynamics in vivo, these approaches still do not permit imaging of the entire brain [9,10,11]. The temporal resolution of these imaging techniques allows for assessments which can be time-locked to specific cognitive and behavioural states and has shed light on many aspects of brain-wide dynamics [12,13,14]. However, due to limitations of anaesthesia head-fixation, or reduced field of view during freely behaving tasks, many questions of brain-wide functional connectivity have remained unanswered. 

These limitations considerably restrict the tasks during which functional connectivity could be studied in animal models. However, there are other markers of neuronal activity which can be used to determine patterns of functional coactivation across a much broader variety of tasks. IEGs are genes which are rapidly transcribed following neuronal activation. While limited in their temporal resolution, several IEGs (such as *c-Fos*, *zif268*/*Erg-1*, and *arc*) have had the time-course of their expression extensively studied with peak expression occurring at specific temporal delays. Regional expression of IEGs can be used as a post hoc histological assessment of the neuronal activity underlying a wide variety of cognitive tasks [15,16,17]. 

By cross correlating regional IEG expression across a group of animals, as real time imaging techniques would correlate across a timeseries, the extent to which fluctuations in the activity of one neuroanatomical region are correlated with the fluctuations in the activity of another region can be assessed. In assessing the correlated activity of all possible pairs of brain regions, coactivation matrices can be generated [18,19]. These coactivation matrices, also known as functional connectivity matrices, form the bases of IEG-based functional connectivity analyses.

Since its first application on a brain-wide scale [17], the use of IEG-based functional connectivity networks as a means of studying global patterns of coactivation has become increasingly prevalent. In recent years, this approach has been used to map functional connectivity underlying a wide variety of behavioural tasks [17,20,21,22,23,24,25,26,27,28,29,30,31,32,33,34,35,36,37,38,39,40,41,42,43,44,45,46,47] (See Supplemental Table S1 for examples). Many of these behavioural tasks rely heavily on the movement of the animal, with this readout often being the primary output of the task. We otherwise would not have been able to access these networks using in vivo neuroimaging techniques, such as functional magnetic resonance imaging. 

IEG-based functional connectivity networks provide unique and important advancement by ushering otherwise inaccessible animal cognitive behavioural tasks into the realm of network neuroscience. In this review, we will discuss technical and analytical considerations which should be kept in mind when designing an experiment using IEG-based functional connectivity networks. Additionally, we will provide examples of the implications of these considerations using an IEG-based network which was generated for this review.

## 2. How to Run the Analysis

### 2.1. Behavioural Tasks 

**Figure 1:**
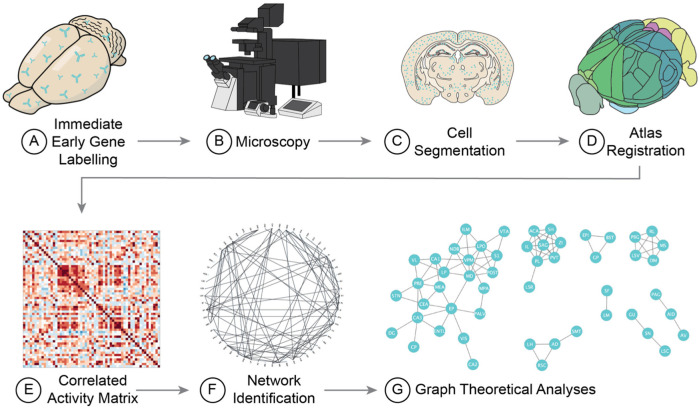
The generation and analysis of functional connectivity datasets from freely behaving animal models with immediate early gene labelling. Following a behavioural task, animals are perfused at a temporal delay corresponding to the peak expression of the IEG of interest. (**A**) In cleared or sectioned tissue, IEGs are labelled and (**B**) imaged across the entire brain. Once images have been collected, (**C**) labelled IEGs are segmented from background. (**D**) Segmented IEG labels are then mapped across the brain by registering the tissue to a standardized brain atlas. (**E**) The regional expression density of IEG labels is then cross correlated across a group of animals to generate a correlated activity matrix. (**F**) In applying thresholds to the correlated activity matrix, binary functional connectivity networks can be identified and (**G**) network topology can be analyzed using graph theory.

Due to the insight that they are able to provide on the dynamics of brain-wide activity in freely moving animals, IEG-based functional connectivity networks have been used to study systems and circuits underlying many behavioural paradigms (See Supplemental Table S1 for distribution of publications across various behavioural tasks and Figure 1 for an example workflow of the procedure). These investigations have yielded insight into many cognitive processes, including the organization of memory storage, circuits underlying prosociality, and the changes in the functional connectome which arise with alcohol dependence [17,30,36].

### 2.2. Histology 

When designing an IEG-based functional connectivity experiment, it is important to consider the benefits and limitations of different IEGs. Notable considerations of the activity proxies commonly used for the purpose of generating functional connectivity networks have been highlighted below (see Supplemental Table S1 for distribution of publications by IEG, and Supplemental Table S2 for an overview of some of the key technical considerations of several commonly used IEGs and other markers of cellular activity). Immunohistochemistry (IHC) or in situ hybridization (ISH) can be performed, although for technical reasons it is much easier to perform IHC labeling especially for brain wide data sets.

*c-Fos*: This IEG was one of the first transcription factors whose induction was shown to be activity-dependent and is expressed through the activation of the CREB/CRE complex. Primarily expressed in the nucleus, the c-Fos protein is evenly distributed throughout the brain and its expression peaks at a temporal delay of 90 min after neuronal activity [48,49]. This temporal delay coincides with a considerably wide tagging window, with detectable protein expression as early as 30 min after induction and remains elevated above baseline until 2 h after neuronal activity [50]. A wide tagging window can be beneficial in that the activity from an entire testing session can be captured, a feature which helped c-Fos become the most studied IEG for IEG-based functional connectivity analyses [51,52,53,54,55]. However, because of this wide tagging window, care must be taken to ensure that experimenters limit the extent to which they cause erroneous neuronal activity.

*zif268/Erg1*: Like c-Fos, the zif268/Erg1 family of IEGs serves as a ubiquitous, rapid, and transient marker of cellular activity with nuclear expression. Additionally, several previously published manuscripts have used this marker for IEG-based functional connectivity analyses [56,57]. However, these similarities aside, there are several key differences between these zif268/Erg1 and c-Fos. Primarily, these two IEGs have similar protein expression and decay rates, but different mRNA temporal expression profiles [50]. Compared to c-Fos, zif268/Erg1 expression density is also much more labile over time, decreasing significantly in some regions with age while remaining stable in others [58]. Furthermore, while present ubiquitously throughout the brain, the density of zif268/Erg1 receptors varies considerably more from region to region than their c-Fos counterparts [59]. However, zif268/Erg1 may provide a unique perspective when applied to network analyses due to its ability to be both up- and down-regulated and the additional dynamic range that this can provide to its expression [60,61].

*Arc*: Activity-regulated cytoskeleton-associated protein, or arc, accumulates at the sites of synaptic activity. As is the case with many IEGs, arc expression varies across the brain [62]. Arc protein regulates the density of AMPA-type glutamate receptors on the surfaces of synapses, to the extent that its expression is tightly associated with the strength of excitatory synapses [63,64]. Thus, this IEG has been the subject of much curiosity in the learning and memory field [65,66,67]. Expression of the arc protein initially peaks at 90 min but can be detected as early as 30 min after neuronal activity, with expression levels remaining elevated from baseline for 4 h after initial expression [68,69,70]. Additionally, a second peak in arc protein expression has been observed at 12 h after neuronal activity [71].

*Homer1a*: Encoding a scaffolding protein within the postsynaptic density, the transcription of the Homer1 gene undergoes a shift following neuronal activation [72]. This activity-dependent shift results in the preferential expression of the short isoform of this mRNA [72]. As such, the expression of short Homer1a protein may be used as a proxy of neuronal activity. Homer1a has been implicated in enabling the necessary plastic changes at the post-synapse during learning and memory processes [73,74]. Homer1a transcripts are detectable within minutes of the onset of neuronal activity, with peak mRNA expression occurring within the nucleus approximately 30 min after neuronal activity, before shifting to cytoplasmic expression by 60 min [75,76,77]. The protein expression time course of this IEG is relatively delayed, with variable reports of peak expression occurring between 2 and 3 h after neuronal activation [78,79]. This is accompanied by a slow decay rate, with Homer1a protein levels remaining elevated above baseline until 8–12 h after neuronal activation [80,81].

*NPAS4*: Neuronal PAS domain protein 4 is a calcium-dependent transcription factor which contributes to the regulation of synapse development in glutamatergic and GABAergic circuits [82]. As such, NPAS4 plays a critical role in regulating the excitatory-inhibitory balance of neural circuits [83]. The expression profile of NPAS4 also varies between excitatory and inhibitory neuron populations, with elevated levels of the transcription factor being detectable for 7.5 h after neuronal activity in excitatory cells, but only for 3 h in inhibitory neuron populations [82,84]. In both cases, elevated NPAS4 can initially be detected one hour after the onset of neuronal activity [82].

### 2.3. Imaging

Major technological advancement which may be contributing to the rise in popularity of brain-wide IEG-based network analyses comes in the form of high-throughput imaging techniques. These techniques, such as high-throughput slide scanning microscopes, light sheet microscopy of cleared tissues, and serial two-photon tomography, have made the imaging of whole-brain tissue sets increasingly efficient and accessible. Using these techniques, we are now able to efficiently process histological markers of neuronal activity across large tissue sets [85,86,87].

### 2.4. Label Segmentation 

Label segmentation is the process through which labels of interest are identified within imaging datasets. While it is certainly possible to perform label segmentation manually, the large-scale nature of the quantification required across many brain regions makes this approach prohibitively time consuming. As such, an automated approach is greatly preferred to facilitate consistent segmentation. There are several open-source options, such as *Ilastik*, *Cell Profiler*, and *3DCellSeg*, which serve as interactive and user-friendly tools for the user-guided segmentation and classification of labels of interest [88,89,90]. 

### 2.5. Image Registration 

Registration of histological images to neuroanatomical datasets on a brain-wide scale can be a very time-intensive and complex task. Commercially available tools (e.g., NeuroInfo [91]) have been developed to facilitate the mapping of labels of interest throughout histological samples. In recent years, open-source atlas registration tools, such as *ClearMap*, *Whole Brain*, *FASTMAP*, *DeepSlice*, *QuickNII*, *SHARCQ*, *CUBIC-Cloud*, and *BrainGlobe*, have also emerged as inexpensive and accessible options to further facilitate the registration of biological images to neuroanatomical atlases [92,93,94,95,96,97,98,99] (See Supplemental Table S3 for examples of open-source and commercially available tools for label segmentation and atlas registration).

Many of these registration techniques enable atlas registration across multiple hierarchical organizations. For example, many tools for registering mouse brains do so based on the *Allen Mouse Brain Atlas* [100,101]. This hierarchically organized atlas has over 500 regions at its most detailed level; however, it also provides 10 other levels of regional organizations which cover these regional subdivisions in increasingly broad organizations. For example, at its most detailed level, the *Allen Mouse Brain Atlas* subdivides the primary motor cortex into cortical layers (MOp1, MOp2/3, MOp5, MOp6a, MOp6b). As we move up in the levels of organizational hierarchy, these layers become grouped together as the primary motor area (MOp), then join a group to form the somatomotor areas (MO). Regions can be organized even more broadly still, as isocortex, then cerebral cortex, then cerebrum. Using this hierarchical organization, experimenters can determine which specific regional organizations best address their experimental questions. The level of registration used in these analyses should be granular enough to address the specific experimental question while still being distinguishable by the experimenter based on the counterstain used during registration. 

### 2.6. Network Analyses 

With regional IEG expression density across multiple animals, brain-wide functional connectivity can now be assessed. In such analyses, functional connectivity reflects the correlated activity of neuroanatomical regions across the brain. When the activity of a pair of regions is highly correlated, they are determined to be functionally connected. In such cases, regions are denoted as nodes and the functional connection that exists between them is considered a vertex. Therefore, functional connectivity matrices can be generated by cross-correlating regional IEG expression density across a group of animals [17]. It is important to consider that while the relationship between regions with highly correlated activity is functional, it does not imply direct structural connections between any pair of regions [102]. 

The primary means of analyzing and comparing IEG-based functional connectivity networks is through a branch of mathematics termed ‘graph theory’. Graph theoretical analyses serve to elucidate the relationships between pairs of nodes, which in the case of IEG-based functional connectivity networks are neuroanatomical regions. Measures of network density and global efficiency can be calculated to assess the nature of network-wide information flow; while measures of clustering coefficients and local efficiency examine the extent to which information processing in localized to specialized subpopulations within networks [103,104,105]. 

Global network parameters allow for us to assess characteristics about the overall organization of functional connectivity across the brain. They also allow for general comparisons to random models to demonstrate the small-world-like network topology which is characteristic of biological systems such as the brain [103,104,106,107,108]. 

On the level of individual regions, it is common to assess the number of functional connections that each region is involved in, or its *degree*, as well as measures indicating its relative importance to the maintenance of efficient informational flow across the network, such as its centrality [105,109,110].

Open-source analysis packages have been developed for the study of each of these metrics, and their relevance to neurobiological systems has been reviewed in great detail. Readers are directed to these comprehensive reviews to explore analysis options for the data generated from a brain-wide IEG activations study [105,111,112,113]. 

## 3. Critical Considerations

### 3.1. Behavioural Tasks 

Many of the behavioural tasks which have been used for IEG-based functional connectivity analyses are centered around a salient cue which yields a reliable behavioural response with minimal drift in strategy, such as a tone during a cued fear conditioning task [22]. There are benefits to selecting a task like this, as reduced variability in cognitive strategy may yield reduced variability in underlying IEG expression patterns [114]. Furthermore, the behavioural responses elicited by these cues are generally sustained for long enough to induce considerable neuronal activity [115]. 

However, that is not to say that this technique cannot be used to examine behaviours which shift over time or tasks which may evoke a wide variety of responses or strategies. By comparing activity markers which reach their maximum express at different temporal delays following the induction of neuronal activity, multiple networks can be generated from the same behavioural task. For example, in tasks with delays between repeated trials, trials could be planned around peaks in the expression profiles of different activity markers. For example, neuronal activity underlying performance of a training trial and a subsequent test trial in a forced alternation Y-maze task could be differentiated using in situ hybridization. With a 30 min intertrial interval, a perfusion 30 min after the test trial would align the training trial with the expected peak in cytosolic *Homer1a* expression (60 min post-activity), and the test trial with the expected peak in nuclear *Homer1a* expression (30 min post-activity). Furthermore, when assessing behaviours with very precise onset and offset times, the activity underlying which could be washed-out using IEG Immunolabeling, can be accessed using fluorescent in situ hybridization [116]. Facilitating both precise temporal tagging and the assessment of activity across multiple timepoints, techniques such as cellular analysis of temporal activity by fluorescence in situ hybridization (catFISH) could provide even greater temporal control over network generation, allowing for comparisons between network activity underlying distinct behavioural states within animals [117]. 

Another means of dealing with variability in behavioural response is to incorporate it into the design of the networks. In tasks with a reliable distribution of cognitive strategies upon which group sizes can be estimated in advance, animals can be assigned to groups based on the strategies that they exhibit during the behavioural task. For example, it had been reported that during a retrieval trial following contextual fear conditioning, 40% of female rats may display a darting response [118,119]. Differences in memory expression between rats exhibiting darting behaviour and those who displayed an increased rate of freezing may coincide with differences in brain wide functional connectivity [114]. Therefore, grouping these animals together may result in a network topology that is not representative of the functional connectivity underlying either darting or non-darting responses. However, if group sizes are calculated with an *a priori* understanding of this variability in behavioral strategy, it can be appreciated as part of the question, rather than an uncontrolled confound. 

While these techniques provide new and exciting means of studying global brain dynamics across a wide variety of tasks, it is important to be mindful of non-task specific sources of activation. Animals from all groups should be habituated to experimenters through repeated handling sessions and to transport between housing and testing facilities. Factors such as time of day and recency of previous cage change should also be controlled for across groups [120,121]. Furthermore, animals across all groups should be treated as similarly as possible during the IEG tagging window to limit the influence of potential confounding variables on the functional connectivity networks being compared. Being cognizant of these potential sources of neuronal activation unrelated to the behavioural task can help to ensure that the functional connectivity networks being studied are robust, reproducible, and representative of the cognitive state being assessed in the task.

### 3.2. Histology 

Consistent tissue sampling from across the entire range of the regions to be included is critical to the generation of IEG-based functional connectivity networks. One of the innovations in histological processing which has enabled this process thoroughly across intact tissue is tissue clearing [122,123,124,125]. Tissue clearing techniques change the optical density of the tissue sample to allow for brain-wide imaging of intact samples. Using this technique, we can eliminate the risk of samples being damaged by sectioning and slide mounting artefacts. Many of these techniques are amenable to immunostaining [86,126,127,128] and have already been used for the generation of IEG-based functional connectivity networks [30,51,52]. Furthermore, with the development of several transgenic IEG reporter lines [129,130,131], even tissue clearing techniques which do not allow for immunolabelling can still be used to image brain wide IEG expression [132].

While tissue clearing presents exciting possibilities in the generation of IEG-based functional connectivity networks, it is important to consider that tissue clearing can be a time-consuming process and requires special imaging platforms. It is important to note however, that these requirements should not serve as a barrier to entry in the use of IEG-based functional connectivity analyses. High quality networks can be generated using serial tissue sectioning combined with standard immunostaining, and microscopy techniques. Regardless of the tissue processing modality, high quality tissue processing is critical in ensuring consistent and clear imaging, as both these will impact all subsequent segmentation and registration efforts.

### 3.3. Imaging 

Any analyses based on image analysis are highly sensitive to the parameters used during image collection. This is particularly true for IEG-based functional connectivity analyses, as imaging parameters will affect both the ability to segment labels of interest as well as the ability to register these images to an atlas.

Brain-wide imaging will often represent a bottleneck in the analysis pipeline. As a result of this it can be tempting to capture lower magnification images which decrease acquisition times as well as data storage demands. It is important to consider the potential limitations associated with the trade-off between image resolution and acquisition speed. It is important to ensure that we are not limiting our ability to accurately segment our labels during image collection. By selecting an imaging magnification which allows for clear identification of labels based on morphological characteristics, we are able to restrict our segmentation to only true cells expressing IEG labels. Furthermore, it is important to maintain a wide dynamic range in the exposure of IEG labels, so as to avoid blurring morphological features of interest.

Imaging magnification is also an important consideration for atlas registration, as it will impact the ability to visually discern the bounds of neuroanatomical regions of interest. In many cases, the differences in cell densities which denote the bounds between these regions are quite subtle and may not be visually apparent at low magnifications. In such instances, the magnification used during image collection will then limit number of regions which can be accurately delineated, thus restricting the regions which can be included in analyses. Additionally, it is important to consider the sampling fraction used during image collection. The majority of open-source and commercially available registration tools use reference atlases composed to serial atlas plates from across a brain [93,133]. These plates exist as sequential 2D planes, rather than as a 3D volume and thus occur over intervals (e.g., 100 µm spacing between plates in the 2011 Allen Mouse Brain Atlas). For optimal atlas registration, images should be gathered from the same 2D places as the target atlas. 

### 3.4. Label Segmentation 

Label segmentation is a critical step in any brain-wide analysis and as such, there are several key considerations which should be kept in mind when establishing a segmentation protocol for an experiment. One such consideration is the extent to which autofluorescence may be influencing segmentation. Across many microscopy techniques, background autofluorescence is highly variable from region to region. This can be influenced by many factors, including regional differences in lipid densities, vascularization, and cell densities [134,135,136]. As such, the segmentation of cells based on intensity relative to a global background may result in poor segmentation in areas of high local autofluorescence. Comparatively, segmentation techniques which detect objects based on local intensity are better able to adapt to regional differences in background autofluorescence [137]. For this reason, the segmentation of cells expressing the IEG label of interest simply based on a global intensity threshold may be sufficient for individual regions but is rarely preferable to segmentation based on local intensity thresholds on a brain-wide level.

Another important factor to consider is the fluorescent intensity range of IEG expression. Given the wide temporal expression profile of IEGs, there exists a wide range of fluorescent intensities in the distribution of IEG+ cells. It is important that the range of fluorescent intensities that the experimenter deems to be indicative of an IEG+ cell remains consistent across all images. Doing so manually presents ample opportunity for experimenter drift, which could drastically impact the results of the experiment.

Thirdly, segmentation can often be further complicated by the presence of artefacts from histological processing. In such cases, nonspecific staining, bubbles, folds, tears and debris may introduce noise to images. If not carefully considered, these artefacts can often fall within the intensity range of the segmentation protocol. Therefore, it is important that such protocols are able to consider the morphology of segmented object to classify objects as a true IEG label or noise.

These factors combined; the use of semi-automated segmentation tools can improve the consistency of this process within an IEG-based network analysis experiment. With tools for identifying labels of interest based on local intensity values, the ability to consistently examine labels within strict intensity bands, and the capability to differentiate true signal from noise based on label morphology; semi-automated segmentation tools can greatly facilitate the consistent segmentation and classification of IEGs throughout the whole brain. 

### 3.5. Image Registration 

The crux of the generation of IEG-based functional connectivity networks is in the registration of labeled tissue to a neuroanatomical atlas. While in many cases, these atlases represent either the ideal representative brain or a average brain derived from many imaging datasets, it is important to consider than not all brains are identical. This is particularly true after histological processing, which can impact the shape and size of the brain. Accordingly, considerable care and attention must be taken during this process, both in terms of node selection and the accuracy of the registration itself.

While standard neuroanatomical atlases often contain hundreds of regions, it is important to consider how many of these regions can be realistically delineated manually without an abundance of region-specific counterstains. There exists a delicate interplay between the level of detail which can be registered using a single counterstain and the reproducibility of atlas registration. Hierarchical atlas organization can assist users in selecting a list of nodes which is both all-encompassing and at a level which is feasible for accurate and reliable delineation based on the counterstain being used.

Another important consideration while performing atlas registration is the suitability of the registration technique for the images being registered. With a wide variety of atlas registration techniques ranging from semi-automatic to largely automatic, the choice of technique can significantly impact the ability to accurately register images. While often significantly faster, many largely automatic registration techniques provide only minimal opportunity for user intervention. With certain pairings of histological protocols and registration tools, such as *iDISCO* and *ClearMap*, this works very well and greatly facilitates the registration process [92]. However, many histological protocols have processes with effects which cause tissue shrinkage [138,139,140,141]. This shrinkage is often uneven, and as a result, tissue will no longer align properly with a superimposed neuroanatomical atlas [133]. Furthermore, even when using complimentary histological protocols and registration techniques, atlases are often developed using specific strains, sexes, and ages; and deviating from the standards used during development may result in slight variations in regional boundaries [142]. Taken together, in most cases it is optimal to have some form of user intervention in the form of a semi-automated image registration pipeline. In many such pipelines, there is an initial atlas transform based on the gross morphology of the target image followed by free-form or correspondence point-based morphing of the atlas to align with the target image [93,94]. While slower than a fully automatic registration protocol, semi-automated registration allows the experimenter to manually account for tissue abnormalities and ensure to the best of their ability that regions of interest are accurately delineated. 

### 3.6. Network Analyses 

#### 3.6.1. Group Size 

**Figure 2:**
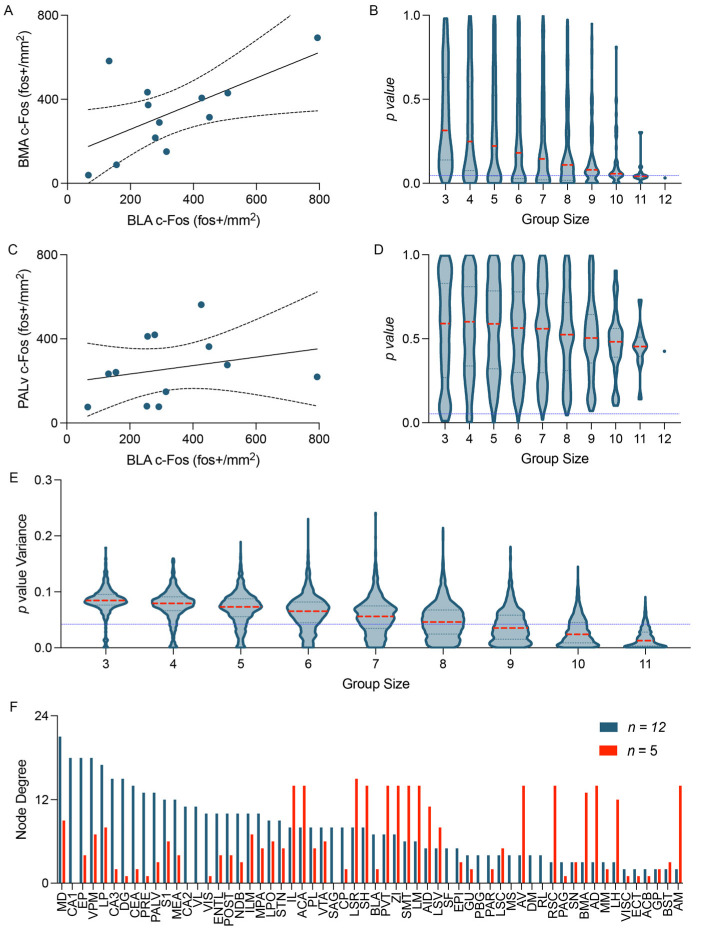
The influence of group size on IEG-based functional connectivity analyses. Regional c-Fos expression density was cross-correlated across 60 neuroanatomical regions (See Supplemental Table S4 for full list of region names and abbreviations) in a group of *n* = 12 mice. The activity of the BLA was significantly correlated with the activity of the BMA (**A**; Pearson *r* = 0.6193; *p* = 0.0318) but did not correlate with the activity of the PALv (**C**; Pearson *r* = 0.2539; *p* = 0.4258). (**B**,**D**) The statistical significance of these correlations was assessed across all possible combinations of these 12 mice in groups of *n* = 3 to *n* = 11. (**E**) The variance of these distributions was plotted for every possible correlation across the entire dataset of 60 neuroanatomical regions. (**F**) The network with the median variance in *p* value of all possible combinations of 5 mice displayed drastically altered global degree distribution compared to the the network constructed from the group of 12 mice. All data is presented as (**A**,**C**) linear regression line ± 95%CI, (**B**,**D**) mean ± 95%CI, and (**E**) median ± quartile ranges. See  Supplemental Table S5 for data used to generate these figures.

It is important to consider the effect of group size in this analysis. As the IEG-based functional connectivity analyses are based on the correlation of IEG expression density within a group, group sizes should be sufficient to yield robust correlations without effects being driven by outliers. Correlation analyses produced from small group sizes are vulnerable to outliers that can drastically change the strength, significance or even direction of the relationship between variables [143,144,145,146]. It is critical to have sufficiently large group sizes to minimize spurious correlations as this will fundamentally change the topography of the network that is generated. To illustrate the consequences of performing this analysis with insufficient group sizes, we analyzed a functional connectivity network using subsets of the total sample size. We first generated pairwise correlation matrices by cross-correlating c-Fos expression density in 60 neuroanatomical regions across a group of 12 mice following contextual memory recall. As expected, c-Fos expression density was found to be significantly correlated between some pairs of regions (basolateral amygdala —basomedial amygdala; Figure 2A), but not significantly correlated between others (basolateral amygdala—ventral pallidum; Figure 2C). Using this dataset, we then produced subsets of mice to examine the impact of sample size on this correlation matrix. Mice were systematically removed from the analysis and correlation matrices were recalculated to obtain networks of all possible combinations of mice for group sizes between *n* = 3 to *n* = 12. As group size decreased, correlations which had been statistically significant across larger samples sizes showed increased occurrence of statistically nonsignificant correlations, or “false negative” results (Figure 2B). Furthermore, when examining a correlation which had previously been not statistically significant, there was an increased rate of occurrence of statistically significant, or “false positive”, correlations (Figure 2D). The variance of the *p* value distribution was calculated for each pair of regions across same-sized networks. As group sizes increased, the mean variability of the *p* values decreased, indicating that the statistical predictability of each correlation became more consistent with increased group size (Figure 2E). This variability was further reflected in the distribution of the connections within the network. When comparing the regional degree, we see fundamental differences in the organization of the network between the full network (*n* = 12) and the network with the median *p* value variance at a smaller group size (*n* = 5; Figure 2F). These differences in organization would have considerable impact on the interpretations that can be formed from these networks. Therefore, it is crucial that small group sizes are avoided when designing such experiments.

#### 3.6.2. Network Thresholding 

When conducting graph theoretical analyses, coactivation matrices can either be analyzed as weighted networks or binary, unweighted networks [147]. In the field of IEG-based functional connectivity networks, weighted network analyses—wherein each correlation retains its weight in the form of the magnitude of the correlation coefficient—are rarely applied quantitatively. Most studies using this approach to assess network properties within this domain will filter their matrices by statistical significance but refrain from binarizing these plots. From these networks, it is common to see descriptive comparisons of trends in the correlation magnitudes between pairs of regions across conditions [42].

**Figure 3:**
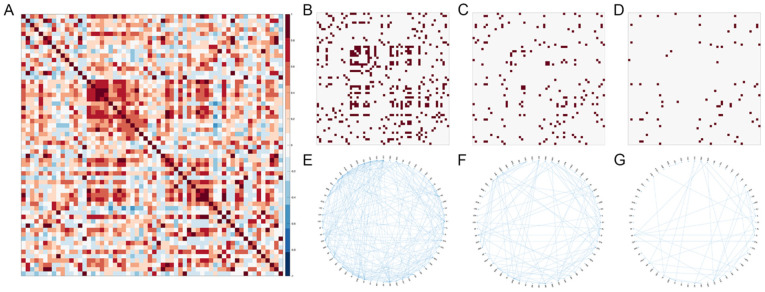
The influence of network threshold on binary network topology. (**A**) Unweighted pairwise correlation matrix denoting the correlated activity between all pairs of neuroanatomical regions in a cohort of *n* = 12 mice. Each row and column represent a distinct neuroanatomical region, with the intersection of each row and column representing the magnitude of the correlated c-Fos expression density between regions. Thresholds of α ≤ 0.05 (**B**,**E**), α ≤ 0.005 (**C**,**F**), and α ≤ 0.0005 (**D**,**G**), were applied to binarize the correlation matrix. Binarized matrices are represented as adjacency matrices (**B**–**D**) and as circle plots (**E**–**G**). See Supplemental Table S4 for full list of regions. See  Supplemental Table S5 for data used to generate these figures.

More commonly, regional coactivation matrices are binarized for unweighted network analyses. It is not uncommon to consult a critical values table and filter by the Pearson’s correlation coefficient corresponding to an alpha value of 0.05 for a particular group size [17]; however, it is important to demonstrate that the results described in subsequent network analyses are not being influenced by the thresholding parameters. As demonstrated in Figure 3, many of the pertinent features of network topology remain consistent across various thresholding parameters. However, measures of global density and efficiency are sensitive to these thresholds and between group differences may not consistently hold true. Therefore, it is critical that findings are assessed at multiple thresholds to ensure that results are robust and not subject to experimenter bias.

*Positive and Negative correlations*: When considering network thresholding, it is also important to consider that statistically significant correlations can occur in both positive and negative directions. Often, anticorrelations are excluded from analyses or their absolute value is studied; however, there is considerable debate about the justification behind these exclusions or adjustments [148,149,150,151]. While present in much lower proportions relative to the density of positive correlations in functional connectivity networks [17,20], their presence is still part of a biological network and thus their exclusion or modification thus obscures the interpretations which can be gleamed from such analyses. One potential interpretation of anticorrelations in functional connectivity is that these relationships might indicated shifting between network states, such as the transition from the classical patterns of brain activity during rest versus brain activity while engaged in focused cognitive processes [150]. Future studies would benefit from further consideration of the relationships being represented in anticorrelated datasets.

## 4. Future Directions

In its current state, brain-wide IEG-based functional connectivity mapping has many benefits, but it is important to acknowledge the means through which it can be improved upon. Many IHC-based approaches are limited by wide protein expression time courses, drastically reducing the temporal specificity of the approach. However, the temporal resolution of ISH is much narrower and in some scenarios may be a better choice. While ISH may be more complicated to apply to a large brain-wide scale than IHC techniques, several recent advances in the technique have made this application more feasible [152,153]. 

Additionally, several transgenic activity reporter lines are now available that leverage IEG promoters to fluorescently tag active neurons [130,154,155,156,157,158,159,160,161]. For more information on these approaches, the use of these genetic manipulations for accessing activated neurons has been reviewed extensively by DeNardo and Luo [162]. These transgenic lines may be useful for brain wide activity mapping studies, but care should be taken to ensure that the labeling is specific to the cell type of interest and widespread in the sense that it is able to be expressed in all regions of interest. This requires careful validation of expression throughout each of the regions of interest. 

Finally, bringing awareness to the careful considerations which should be made while planning and conducting these analyses will contribute to the advancement of this technique. With improved awareness of the benefits and limitations of this technique, there is hope that it will be responsibly applied to appropriately designed studies to provide interesting new theories and developments in the neurosciences.

## 5. Conclusions

Underlying cognitive processes are brain-wide patterns of activity. Traditional approaches to measuring this functional connectivity have established many analysis techniques and have opened many paths towards the goal of better understanding the brain. However, technical limitations often restrict the ability of these approaches to be applied to certain behavioural tasks in animal models. IEG-based functional connectivity networks provide a powerful approach to assess these otherwise unanswerable questions.

While the ability to gleam insight into functional connectivity in freely behaving animal models fosters the assessment of brain-wide dynamics across a wide range of otherwise inaccessible questions, it is important to acknowledge the caveats of this approach. In correlating across a group, rather than across a timeseries as is typical of other neuroimaging techniques, functional connectivity is assessed at a group level rather than at the level of the individual. Therefore, this technique will yield network analyses that are only as homogenous as the patterns of brain wide activity of the members of the groups being analyzed. Due to this caveat, we propose that IEG-based functional connectivity analyses should not be viewed as a replacement to other neuroimaging techniques, but rather as a complementary approach which expands our ability to infer functional connectivity during behavioural episodes and can identify regions for other, more temporally dynamic, techniques to explore.

Furthermore, it is important to consider the limitations of the interpretations which can be made developed from these analyses. In the absence of a targeted stimulation to initiate activity at a seed region, IEG-based functional connectivity networks should not be used to interpret causal relationships between pairs of regions. Rather, these analyses provide the ability to parse functional relationships between pairs of regions on a brain-wide scale, providing insight into the distribution of activity and highlighting key regions. The benefit of such an approach may be in identifying relationships between distant pairs of target regions for future investigation.

In the present review, we have presented several critical considerations which should be kept in mind when conducting IEG-based functional connectivity analyses. We have also presented examples of the implications of these considerations. We hope that these points will help to guide the statistically responsible and reproducible use of this technique moving forward and as a guide for anyone who wishes to incorporate these techniques in their quest to better understand the brain. 

## 6. Methods

### 6.1. Mice 

8-week-old C57BL/6J mice (JAX) were used for all experiments. Mice (*n* = 12) were housed in standard cages with three to five mice per cage and free access to food and water. The room lighting was maintained on a 12 h/12 h light/dark cycle (light on 8:00–20:00). Behavioural testing was conducted during the light cycle phase. Experiments were conducted in accordance with the policies and guidelines of the Canadian Council on Animal Care and were approved by the University of Calgary Animal Care Committee.

### 6.2. Contextual Fear Conditioning 

Mice were trained in contextual fear conditioning. Training was conducted in sound-attenuated chambers with grated floors through which shocks (0.5 mA; 2 s) were delivered (Ugo Basile, Germonio, Italy). Mice were allowed 5 min to acclimate to the chamber before receiving their first shock. In total, 5 shocks were delivered over the course of a single 20 min training session. 48 h after training, mice were returned to the conditioning chambers for an 8 min retention test. During this test, no shocks were administered and behavioural was monitored via and overhead, infrared camera in conjunction with an automated tracking software (ANY-maze, Stoelting, Wood Dale, IL, USA). The chambered was cleaned using 70% ethanol and allowed to dry before and after each trial.

### 6.3. Perfusions and Histology 

Mice were transcardially perfused with 0.1 M phosphate-buffered saline (PBS) followed by 4% formaldehyde 90 min after retention testing. Brains were then extracted and postfixed in 4% formaldehyde for 24 h before cryoprotection in 30% *w/v* sucrose solution at 4 °C until no longer buoyant. Serial sagittal sections were cut on a cryostat (Leica Biosystems, Concord, ON, Canada) at 40 µm and stored in 12 series at −20 °C in antifreeze solution. 

Immunohistochemistry: Tissue sections were washed 3 times (10 min per wash) in 0.1 M PBS before being incubated in a primary antibody solution of 1:2000 rabbit anti-c-Fos (RPCA-c-FOS; EnCor Biotechnology, Gainesville, FL, USA), 3% normal goat serum, and 0.3% Triton-X100 for 48 h at room temperature on a tissue shaker. Tissue sections were washed 3 times in 0.1 M PBS before 24 h incubation in 1:500 goat anti-rabbit Alexa Fluor 647 (111-605-003; Jackson Immuno, West Grove, PA, USA) in 0.1 M PBS. Sections were DAPI stained (1:1000, 15 min) before being washed 3 times and mounted to glass slides. Slides were coverslipped with PVA-DABCO mounting medium.

### 6.4. Brain-Wide c-Fos Quantification 

All slides were imaged as a single batch using an Olympus VS120-L100-W slide scanning microscope (Richmond Hill, ON, Canada). Images were collected using a 10× objective with a numerical aperture of 0.40 and a Hamamatsu ORCA-Flash4.0 camera. Labelled c-Fos was imaged using a CY5 filter cube and a 9.00 V lamp at an exposure time of 70 ms. DAPI staining was imaged under the same conditions, but with a DAPI filter cube and an exposure time of 12 ms. Cells expressing a c-Fos label were segmented using the machine learning-based pixel and object classification program, *Ilastik* [88]. Images were further preprocessed using a custom *ImageJ* macro script to yield binary images of segmented c-Fos labels and a mask of evenly spaced grid-points (22 µm spacing) arranged as a mask for each tissue section. Registration of these images based on the DAPI channel to the *Allen Mouse Brain Atlas* using the *R*-based *Whole Brain* software [93] provided regional counts of c-Fos and grid points. Using a Cavalieri-based point counting approach, regional grid points were used to calculate the area of each region and facilitated the assessment of regional c-Fos expression density [29].

### 6.5. Functional Connectivity Network Generation and Analysis 

Functional connectivity analyses were focused to a collection of 60 regions based on our ability to reliably delineate these regions using a DAPI stained images as reference (see Supplementary Data S1 for list of regions). The density of regional c-Fos expression was cross-correlated within each group to generate pairwise correlation matrices. Correlations were filtered by statistical significance and a false discovery rate of 5% [163,164]. All network analyses were conducted on binary adjacency matrices. Correlation matrices were binarized using several pairings of correlation coefficient and alpha value thresholds. The stringency of these thresholds was varied to demonstrate the potential influence of thresholding parameters on subsequent network analyses. From each binary matrix, the neuroanatomical regions were plotted as nodes and connections were drawn between nodes whereby correlations surpassed thresholding parameters. 

### 6.6. Statistical Analyses 

All analyses of functional connectivity networks were conducted using custom MATLAB analyses. Figures were generated using GraphPad Prism (GraphPad Software, San Diego, CA, USA), MATLAB R2020a (The MathWorks Inc., Natick, MA, USA), Cytoscape [165], and Adobe Illustrator 2020.

## Data Availability

The data that support the findings of this study are available in the manuscript or supplementary materials, and available from the corresponding author upon reasonable request. All analysis code used to generate the results reported in the manuscript and instructions on how to use these analyses have been made publicly available in a GitHub repository (https://github.com/dterstege/PublicationRepo/tree/main/Terstege2022D, (accessed on 17 November 2022)).

## Supplementary Materials

The following supporting information can be downloaded at https://www.mdpi.com/article/10.3390/biology12010034/s1, Table S1: Publications using IEG-based functional connectivity analyses; Table S2: Immediate Early Genes; Table S3: Registration and Segmentation Tools; Table S4: Region list for the present IEG-based functional connectivity analyses; Table S5: Data used for the generation of all figures in the present manuscript. References [48,50,59,60,61,68,69,70,71,72,77,78,79,80,81,82,83,84,88,89,90,92,93,94,95,97,98,99,117,166,167,168,169] are cited in the supplementary materials.
