# Supplementary material for: Network Neuroscience Untethered: Brain-Wide Immediate Early Gene Expression for the Analysis of Functional Connectivity in Freely Behaving Animals
Source: Biology (Basel). 2022 Dec 24;12(1):34. doi: 10.3390/biology12010034 (PMC9855808; doi:10.3390/biology12010034)
Supplement: Supplementary file 1 [file biology-12-00034-s001.zip › Supplementary Table S2.pdf]

Supplementary Table S2. Immediate early genes. Temporal expression profiles and other considerations for several common IEGs.

Marker	Expression Peak(s)	Decay Rate	Special Considerations
c-Fos	*mRNA*: 30 minutes after the onset of activity [1].<br><br>*Protein*: 90 minutes after the onset of activity [1].	*mRNA*: Remains elevated until 90 minutes after neuronal activity [1].<br><br>*Protein*: Remains elevated until 2 hours after neuronal activity [1].	One of the first identified activity-dependent transcription factors [2,3].<br><br>Expression patterns may display an adaptive response [4–6].
Zif268/Erg1	*mRNA*: 30 minutes after the onset of activity [1].<br><br>*Protein*: 90 minutes after the onset of activity [1].	*mRNA*: Remains elevated until 2 hours after neuronal activity [1].<br><br>*Protein*: Remains elevated until 2 hours after neuronal activity [1].	Density of Erg1 receptors varies considerably from region to region [7].<br><br>May be both up- and down-regulated [8,9].
Arc	*mRNA*: 15 – 30 minutes after the onset of activity [10].<br><br>*Protein*: 60 – 90 minutes after the onset of activity [11]; second peak at 12 hours [12].	*mRNA*: Remains elevated until 20 minutes after neuronal activity [10].<br><br>*Protein*: Remains elevated until 4 hours after neuronal activity [11].	mRNA expression peak varies from region to region [13].<br><br>Regulates the density of AMPA-type glutamate receptors [14].
Homer1a	*mRNA*: nuclear expression peak 30 minutes after the onset of activity. Cytosolic expression peak 60 minutes after the onset of activity [15].<br><br>*Protein*: 120 - 180 minutes after the onset of activity [16,17]	*mRNA*: Remains elevated until 2.5 hours after the onset of activity [18].<br><br>*Protein*: Remains elevated long after initial induction, with significantly increased protein density detectable even 8 hours later [17].	Homer1 gene preferentially transcribed as its short *Homer1a* isoform following neuronal activity [15].<br><br>Long isoforms (*Homer1b/c*) contribute to the regulation of NMDA and AMPA receptor activity[19,20].
NPAS4	*mRNA*: 1 hour after the onset of activity [21].	*mRNA*: remains elevated until 7.5 hours after neuronal activity in	Differential expression profiles in excitatory vs

		excitatory neurons, but only for 3 hours in inhibitory neuron populations [21]	inhibitory neuron types [22].<br><br>Implicated in the regulation of the excitatory-inhibitory balance of neural circuits [23].

References

1. Zangenehpour, S.; Chaudhuri, A. Differential Induction and Decay Curves of C-Fos and Zif268 Revealed through Dual Activity Maps. *Brain Res. Mol. Brain Res.* **2002**, *109*, 221–225.
2. Morgan, J.I.; Curran, T. Calcium as a Modulator of the Immediate-Early Gene Cascade in Neurons. *Cell Calcium* **1988**, *9*, 303–311.
3. Sagar, S.M.; Sharp, F.R.; Curran, T. Expression of C-Fos Protein in Brain: Metabolic Mapping at the Cellular Level. *Science* **1988**, *240*, 1328–1331.
4. Gall, C.M.; Hess, U.S.; Lynch, G. Mapping Brain Networks Engaged by, and Changed by, Learning. *Neurobiol. Learn. Mem.* **1998**, *70*, 14–36.
5. Maleeva, N.E.; Bikbulatova, L.S.; Ivolgina, G.L.; Anokhin, K.V.; Limborskaia, S.A.; Kruglikov, R.I. Activation of the c-fos proto-oncogene in different structures of the rat brain during training and pseudoconditioning. *Dokl. Akad. Nauk SSSR* **1990**, *314*, 762–764.
6. Maleeva, N.E.; Ivolgina, G.L.; Anokhin, K.V.; Limborskaia, S.A. Analysis of the expression of the c-fos proto-oncogene in the rat cerebral cortex during learning. *Genetika* **1989**, *25*, 1119–1121.
7. Farina, F.R.; Commins, S. Differential Expression of Immediate Early Genes Zif268 and C-Fos in the Hippocampus and Prefrontal Cortex Following Spatial Learning and Glutamate Receptor Antagonism. *Behav. Brain Res.* **2016**, *307*, 194–198.
8. Rockel, J.S.; Bernier, S.M.; Leask, A. Egr-1 Inhibits the Expression of Extracellular Matrix Genes in Chondrocytes by TNFalpha-Induced MEK/ERK Signalling. *Arthritis Res. Ther.* **2009**, *11*, R8.
9. Duclot, F.; Kabbaj, M. The Role of Early Growth Response 1 (EGR1) in Brain Plasticity and Neuropsychiatric Disorders. *Front. Behav. Neurosci.* **2017**, *11*, 35.
10. Guzowski, J.F.; McNaughton, B.L.; Barnes, C.A.; Worley, P.F. Environment-Specific Expression of the Immediate-Early Gene Arc in Hippocampal Neuronal Ensembles. *Nat. Neurosci.* **1999**, *2*, 1120–1124.
11. Lonergan, M.E.; Gafford, G.M.; Jarome, T.J.; Helmstetter, F.J. Time-Dependent Expression of Arc and Zif268 after Acquisition of Fear Conditioning. *Neural Plast.* **2010**, *2010*, 139891.
12. Nakayama, D.; Iwata, H.; Teshirogi, C.; Ikegaya, Y.; Matsuki, N.; Nomura, H. Long-Delayed Expression of the Immediate Early Gene Arc/Arg3.1 Refines Neuronal Circuits to Perpetuate Fear Memory. *J. Neurosci.* **2015**, *35*, 819–830.
13. Khodadad, A.; Adelson, P.D.; Lifshitz, J.; Thomas, T.C. The Time Course of Activity-Regulated Cytoskeletal (ARC) Gene and Protein Expression in the Whisker-Barrel Circuit Using Two Paradigms of Whisker Stimulation. *Behav. Brain Res.* **2015**, *284*, 249–256.
14. Guzowski, J.F.; Lyford, G.L.; Stevenson, G.D.; Houston, F.P.; McGaugh, J.L.; Worley, P.F.; Barnes, C.A. Inhibition of Activity-Dependent Arc Protein Expression in the Rat Hippocampus Impairs the Maintenance of Long-Term Potentiation and the Consolidation of Long-Term Memory. *J. Neurosci.* **2000**, *20*, 3993–4001.
15. Bottai, D.; Guzowski, J.F.; Schwarz, M.K.; Kang, S.H.; Xiao, B.; Lanahan, A.; Worley, P.F.; Seeburg, P.H. Synaptic Activity-Induced Conversion of Intronic to Exonic Sequence in Homer 1 Immediate Early Gene Expression. *J. Neurosci.* **2002**, *22*, 167–175.
16. Diering, G.H.; Nirujogi, R.S.; Roth, R.H.; Worley, P.F.; Pandey, A.; Huganir, R.L. Homer1a Drives Homeostatic Scaling-down of Excitatory Synapses during Sleep. *Science* **2017**, *355*, 511–515.
17. Zhang, G.-C.; Mao, L.-M.; Liu, X.-Y.; Parelkar, N.K.; Arora, A.; Yang, L.; Hains, M.; Fibuch, E.E.; Wang, J.Q. In Vivo Regulation of Homer1a Expression in the Striatum by Cocaine. *Mol. Pharmacol.* **2007**, *71*, 1148–1158.
18. Lin, R.; Learman, L.N.; Bangash, M.A.; Melnikova, T.; Leyder, E.; Reddy, S.C.; Naidoo, N.; Park, J.M.; Savonenko, A.; Worley, P.F. Homer1a Regulates Shank3 Expression and Underlies Behavioral Vulnerability to Stress in a Model of Phelan-McDermid Syndrome. *Cell Rep.* **2021**, *37*, 110014.

19. Cingolani, L.A.; Vitale, C.; Dityatev, A. Intra- and Extracellular Pillars of a Unifying Framework for Homeostatic Plasticity: A Crosstalk between Metabotropic Receptors and Extracellular Matrix. *Front. Cell. Neurosci.* **2019**, *13*, 513.
20. Bertaso, F.; Roussignol, G.; Worley, P.; Bockaert, J.; Fagni, L.; Ango, F. Homer1a-Dependent Crosstalk between NMDA and Metabotropic Glutamate Receptors in Mouse Neurons. *PLoS One* **2010**, *5*, e9755.
21. Spiegel, I.; Mardinly, A.R.; Gabel, H.W.; Bazinet, J.E.; Couch, C.H.; Tzeng, C.P.; Harmin, D.A.; Greenberg, M.E. Npas4 Regulates Excitatory-Inhibitory Balance within Neural Circuits through Cell-Type-Specific Gene Programs. *Cell* **2014**, *157*, 1216–1229.
22. Coutellier, L.; Beraki, S.; Ardestani, P.M.; Saw, N.L.; Shamloo, M. Npas4: A Neuronal Transcription Factor with a Key Role in Social and Cognitive Functions Relevant to Developmental Disorders. *PLoS One* **2012**, *7*, e46604.
23. Bloodgood, B.L.; Sharma, N.; Browne, H.A.; Trepman, A.Z.; Greenberg, M.E. The Activity-Dependent Transcription Factor NPAS4 Regulates Domain-Specific Inhibition. *Nature* **2013**, *503*, 121–125.
