# Supplementary material for: Network Neuroscience Untethered: Brain-Wide Immediate Early Gene Expression for the Analysis of Functional Connectivity in Freely Behaving Animals
Source: Biology (Basel). 2022 Dec 24;12(1):34. doi: 10.3390/biology12010034 (PMC9855808; doi:10.3390/biology12010034)
Supplement: Supplementary file 1 [file biology-12-00034-s001.zip › Supplementary Table S3.pdf]

**Supplementary Table S3. Registration and Segmentation Tools.** A selection of open-source and commercially available tools for label segmentation and histological image registration in neuroanatomical datasets.

| **Software** | **Type** | **Open-Source/Commercial** | **Website/Publication** |
|---|---|---|---|
| *Ilastik* [1] | Label Segmentation | Open-Source | https://www.ilastik.org |
| *CellProfiler* [2] | Label Segmentation | Open-Source | https://cellprofiler.org |
| *3DCellSeg* [3] | Label Segmentation | Open-Source | https://github.com/AntonotnaWang/3DCellSeg |
| *Olympus CellSens TruAI* | Label Segmentation | Commercial | https://www.olympus-lifescience.com/en/landing/truai/ |
| *FASTMAP* [4] | Image Registration | Open-Source | https://github.com/dterstege/FASTMAP |
| *ClearMap* [5] | Image Registration | Open-Source | https://christophkirst.github.io/ClearMap2Documentation/html/home.html |
| *Whole Brain* [6] | Image Registration | Open-Source | https://www.wholebrainsoftware.org |
| *CUBIC-Cloud* [7] | Image Registration | Open-Source | https://cubic-cloud.com |
| *DeepSlice* [8] | Image Registration | Open-Source | https://github.com/PolarBean/DeepSlice/ |
| *QuickNII* [9] | Image Registration | Open-Source | https://github.com/HumanBrainProject/QuickNII |
| *SHARCQ* [10] | Image Registration | Open-Source | https://github.com/wildrootlab/SHARCQ |
| *NeuroInfo* | Image Registration | Commercial | https://www.mbfbioscience.com/products/neuroinfo |

## References


1. Berg, S.; Kutra, D.; Kroeger, T.; Straehle, C.N.; Kausler, B.X.; Haubold, C.; Schiegg, M.; Ales, J.; Beier, T.; Rudy, M.; et al. Ilastik: Interactive Machine Learning for (Bio)Image Analysis. *Nat. Methods* **2019**, *16*, 1226–1232.
2. McQuin, C.; Goodman, A.; Chernyshev, V.; Kamentsky, L.; Cimini, B.A.; Karhohs, K.W.; Doan, M.; Ding, L.; Rafelski, S.M.; Thirstrup, D.; et al. CellProfiler 3.0: Next-Generation Image Processing for Biology. *PLoS Biol.* **2018**, *16*, e2005970.
3. Wang, A.; Zhang, Q.; Han, Y.; Megason, S.; Hormoz, S.; Mosaliganti, K.R.; Lam, J.C.K.; Li, V.O.K. A Novel Deep Learning-Based 3D Cell Segmentation Framework for Future Image-Based Disease Detection. *Sci. Rep.* **2022**, *12*, 342.
4. Terstege, D.J.; Oboh, D.O.; Epp, J.R. FASTMAP: Open-Source Flexible Atlas Segmentation Tool for Multi-Area Processing of Biological Images. *eNeuro* **2022**, *9*, ENEURO.0325-21.2022.
5. Renier, N.; Adams, E.L.; Kirst, C.; Wu, Z.; Azevedo, R.; Kohl, J.; Autry, A.E.; Kadiri, L.; Umadevi Venkataraju, K.; Zhou, Y.; et al. Mapping of Brain Activity by Automated Volume Analysis of Immediate Early Genes. *Cell* **2016**, *165*, 1789–1802.
6. Fürth, D.; Vaissière, T.; Tzortzi, O.; Xuan, Y.; Märtin, A.; Lazaridis, I.; Spigolon, G.; Fisone, G.; Tomer, R.; Deisseroth, K.; et al. An Interactive Framework for Whole-Brain Maps at Cellular Resolution. *Nat. Neurosci.* **2018**, *21*, 139–149.
7. Mano, T.; Murata, K.; Kon, K.; Shimizu, C.; Ono, H.; Shi, S.; Yamada, R.G.; Miyamichi, K.; Susaki, E.A.; Touhara, K.; et al. CUBIC-Cloud Provides an Integrative Computational Framework toward Community-Driven Whole-Mouse-Brain Mapping. *Cell Rep Methods* **2021**, *1*, 100038.
8. Carey, H.; Pegios, M.; Martin, L.; Saleeba, C.; Turner, A.; Everett, N.; Puchades, M.; Bjaalie, J.; McMullan, S. DeepSlice: Rapid Fully Automatic Registration of Mouse Brain Imaging to a Volumetric Atlas. *bioRxiv* 2022.
9. Puchades, M.A.; Csucs, G.; Ledergerber, D.; Leergaard, T.B.; Bjaalie, J.G. Spatial Registration of Serial Microscopic Brain Images to Three-Dimensional Reference Atlases with the QuickNII Tool. *PLoS One* **2019**, *14*, e0216796.
10. Lauridsen, K.; Ly, A.; Prévost, E.D.; McNulty, C.; McGovern, D.J.; Tay, J.W.; Dragavon, J.; Root, D.H. A Semi-Automated Workflow for Brain Slice Histology Alignment, Registration, and Cell Quantification (SHARCQ). *eNeuro* **2022**, *9*, ENEURO.0483-21.2022.
